# Screening of Novel Source for Genistein by Rapid and Sensitive UPLC-APCI-TOF Mass Spectrometry

**DOI:** 10.1155/2021/5537917

**Published:** 2021-03-16

**Authors:** Aparna Bettaiah, Hema Bommanamane Prabhushankar

**Affiliations:** Department of Biotechnology, Sri Jayachamarajendra College of Engineering, JSS Science and Technology University, JSS TI Campus, Mysuru, Karnataka 57006, India

## Abstract

Genistein has been shown to have a broad spectrum of health advantages. Only legumes were reported to have a significant amount of genistein with the highest concentration in Soybean. Soybean was found to cause allergies in children with atopic dermatitis and in adults. Limited food sources have hindered the use of genistein in daily diets, medications, and nutraceuticals. The main objective of the current research work was to discover the novel source for genistein by the simple method of extraction and quantification. Genistein was extracted by solid-liquid extraction technique. Extraction parameters were optimized by a single factor test. Identification and quantification of genistein from the selected seeds of Apiaceae were carried out using UPLC-APCI-TOF-MS. UPLC-APCI-TOF-MS method was successfully developed, validated (linearity (R^2^ = 0.999), precision (R.S.D. <5%), and accuracy (107.23%)), and used for the study. Remarkably, a high concentration of the genistein (811.57 *μ*g/g) was found in the *Cuminum cyminum*. Solvent mixture (50 mL Methanol+25 mL Dimethyl sulphoxide+25 mL Water (*v*/*v*/*v*)), temperature (80°C), and time (1 h) were found to be the optimum extraction conditions. The concentration of genistein before optimization was 226.67 *μ*g/g and after optimization is 811.57 *μ*g/g. This shows the efficiency of the extraction method in the extraction of genistein without the need for hydrolysis. Novel source for genistein is identified in regular human food can be consumed in a regular diet which increases wellness of human health along with enhancing the taste of the food. The developed extraction method coupled with high throughput, sensitive, and selective UPLC-APCI-TOF-MS technique facilitates rapid quantification (8 minutes of run time) without primary purification of complex extract.

## 1. Introduction

Primary prevention of chronic and degenerative diseases through lifestyle interventions is the best choice for preventing morbidity and mortality. Diet is one of the important contributing factors for many chronic diseases [1]. One particular class of dietary compounds that has gained much attention because of their reported antiproliferative activity are phytoestrogens. One in eight white women in the United States can expect to develop breast cancer in their lifetime; in Japanese and Chinese women living in Asia, this probability is about 5 times lower [2]. This was found to be associated with the consumption of phytoestrogen-rich foods particularly soy products and legumes, which is higher in Asia than in the Western world [3].

Genistein (4′, 5, 7-trihydroxyisoflavone) is often referred to as phytoestrogen, which is a plant-derived nonsteroidal estrogen mimic [4], usually present as glucosides in plants which are hydrolysed to release the aglycones by the intestinal enzyme, glucosidases [5]. Genistein is known for its anticancer activity. Cell line studies conducted on colorectal cancer cells (HCT-116, LoVo & HT-29) [6, 7], pancreatic cancer (Mia-PaCa2 & PANC-1) [8], Human breast cancer (MCF-7, SK-BR-3, MDA-MB-231 & ZR-75-1) [9, 10], Human oesophageal cancer cell lines (Eca-109, EC9706 & CaES-17) [11], and Prostate cancer (PC3) [12] have shown genistein as the potent inhibitor of cancer cell proliferation. However, a few *in vitro* studies have reported genistein to induce proliferation in breast cancer cells [13, 14], but none of the clinical trial witnesses this. The role of genistein in relation with the proliferation of breast cancer cells mainly depends on the dosage of genistein, time of exposure, and relative ratios of *α*/*β* ER isoforms [15]. Epidemiological studies have shown that the incorporation of genistein in regular diet has shown to have a wide range of significant health advantages. This includes many hormone-dependent disorders, aging-related health issues (osteoporosis and postmenopause symptoms), and cardiovascular diseases [4]. Recently conducted clinical trials on postmenopause women have showed promising results in treating osteoporosis, hot flushes, and other postmenopause symptoms [16–18]. Clinical trial results revealed genistein as the safe compound for consumption and not found to have negative effects on either breast cell proliferation or on endometrial thickness [19]. Genistein exerts its anticancer effects through its pleiotropic molecular mechanism by acting upon cell cycle, cell apoptotic processes, angiogenesis, invasion, and metastasis through various molecular mechanism(s) of action. The potency of genistein to act so is primarily due to specific action of genistein on inhibition of PTKs (EGFR/VEGFR/Her2), signalling pathways Akt, NF-*κ*B, MMPs, Bax/Bcl-2, and interaction with ER*α*b [15].

On the other hand, genistein was experimentally proven as the most active flavonoid which has a capacity to stimulate enzymatic cleave of DNA ~10 fold [20], and evidences also suggest genistein as an effective remedy against intestinal parasites such as the common liver fluke and pork trematode [21, 22].

Genistein content of commonly eaten food sources has been evaluated and found substantial amounts only in leguminous plants with the highest concentrations in Soybean [23]. Research studies have shown that Soybean consists of antinutritional factors such as protease inhibitors, lectins, tannins, and phytates, and these molecules were found to contribute to the disturbed digestion process and absorption in the small intestine [24], and Soybean was also reported to cause allergies (anaphylaxis) [25]. The broad spectrum of health advantages and limited food sources for genistein has stimulated much interest in the investigation of a new principal food source for genistein in human diet.

Many methods have been reported for quantification of genistein including UV-Visible spectrophotometric method, thin layer chromatography (TLC), high-performance liquid chromatography (HPLC), and gas chromatography [26–28]. These analytical methods had considerable disadvantages such as lack of specificity, sensitivity, and long run time. Using UPLC-APCI-TOF-MS, the analysis time demand can be greatly reduced as fewer sample preparation steps are required and is capable of detecting desired components under or around the ppb level. Although UPLC-tandem mass spectrometry has been used successfully for the determination of genistein in *Genista tinctoria* [29] and in Mung bean [30], to date, there is no ultrapressure liquid chromatography–mass spectrometry (UPLC–MS) method available for the estimation of genistein in different plant seeds of Apiaceae. Here, in this study, the UPLC-APCI-TOF-MS technique was employed with a simple method of extraction for the analysis of genistein in Apiaceae. The proposed method has significant advantages over earlier reported methods like shorter run time, comparatively less amount of solvent consumption, wider linearity range with a more sensitivity.

## 2. Materials and Methods

### 2.1. Chemicals and Reagents

Analytical standard genistein is purchased from Sigma-Aldrich (U.S.). HPLC grade Formic acid, Acetonitrile, Ethanol, Methanol, and Dimethyl Sulphoxide (DMSO) were procured from Merck (Darmstadt, Germany). Milli-Q water was generated using a Milli-Q purification unit (Millipore, Bangalore, India) and used throughout the study.

### 2.2. Sample Collection and Preparation

Seven seed samples, namely *Coriandrum sativum*, *Apium graveolens*, *Cuminum cyminum*, *Foeniculum vulgare*, *Nigella sativa*, *Carum carvi*, and *Anethum graveolens*, were purchased from local supermarket (Mysore, Karnataka); voucher specimen was numbered and deposited in the Dept. of Biotechnology, SJCE, Mysuru. Seeds were milled to fine powder, sieved (80 mesh), and refrigerated in airtight container at 4°C until analysis.

### 2.3. Preparation of Seed Extracts

All seven pulverized seed samples (1 g) were extracted with 100 mL of 90% Methanol at 70°C for 1 h with continuous shaking in hot water bath shaker. Filtered extract solutions were concentrated to 10 mL by keeping it in a hot air oven at 70°C (initial extraction conditions). This extract solution was directly injected to the UPLC-APCI-TOF-MS system for screening and quantification of genistein.

### 2.4. Chromatographic and Mass Spectrometric Conditions

The analytical separation and quantification analysis were performed on a Waters ACQUITY UPLC system coupled with APCI-TOF mass spectrometer. Waters acquity UPLC system (Waters Corporation, Milford, USA) consisted of column manager, binary solvent manager, and sample manager to facilitate solvent and sample delivery. Gradient elution method was followed for better separation, where mobile phase A consisted of 0.1% Formic acid in water and mobile phase B was Acetonitrile.

The analytes were separated by the following gradient (minutes, % mobile phase A): 0 min 98% A, 4 min 2% A, 6 min 2% A, 7 min 9% A, and 8.0 min 98% A, and delivered with a flow rate of 0.2 mL/min on a ACQUITY UPLC BEH C_18_ (1.7 *μ*m 1.0 × 50 mm) maintained at 50°C. The sample manager was maintained at 5°C, and the typical injection volume of extract was 2 *μ*L. The run time was fixed as 8 min to allow full chromatographic separation which is necessary to avoid matrix effects. The concentration of analyte and standard were detected using a Waters mass spectrometer (Waters Corporation, Milford, USA) equipped with time of flight (TOF) as mass analyser and atmospheric pressure chemical ionization (APCI) as ionizing source. The quantification analysis was achieved by operating the mass spectrometer in negative ion APCI mode, and mass spectrometry data was represented as base peak intensity (BPI) chromatogram. MS system was operated under nebulizer pressure 60 psi, vaporizer temperature 475°C, sheath gas flow rate 90 arbitrary units, discharge current 5 V, capillary temperature 170°C, capillary voltage –30 kV, and electrospray voltage 4.5 kV.

### 2.5. Identification and Quantification

Isoflavone genistein in selected plant seed samples was identified by comparing the retention time and mass spectra of unknown peaks with the retention time and mass spectra of the reference standard. The quantification of genistein in test samples was done using an external calibration curve of standard. The standard solution of genistein at varying concentrations was injected into UPLC-APCI-TOF-MS, and calibration curve was constructed by plotting peak area as *x*-axis vs. concentration (*μ*g/g) as *y*-axis.

### 2.6. Optimization of Extraction Conditions by Single Factor Test

There are several factors influencing the extraction yield, of which the key factors are extraction solvent, extraction time, and extraction temperature. One of the objectives of this work was to propose suitable method conditions to extract maximum genistein from the sample. Optimization studies were carried on by following a method called single factor test, which is performed by varying one factor/parameter at a time by keeping the rest of the parameters constant.

#### 2.6.1. Effect of Solvent Composition on the Extraction Yield of Genistein

Genistein is generally soluble in polar organic solvents, and subsequently, the concentration of these solvents is also very important in extracting the maximum amount of genistein from the sample. Initial extraction was performed by taking 1 g of sample in 100 mL of solvent at 70°C for 1 h. Extraction was carried out using six different solvent mixtures (1) Methanol : Water (90 : 10, *v*/*v*), (2) Methanol : Water : Dimethyl sulphoxide (90 : 5 : 5 *v*/*v*), (3) Ethanol : Water (70 : 30, *v*/*v*), (4) Ethanol : Water : Dimethyl sulphoxide (70 : 25 : 5, *v*/*v*), (5) Acetonitrile : Water (58 : 42, *v*/*v*), and (6) Acetonitrile : Water : Dimethyl sulphoxide (58 : 37 : 5, *v*/*v*). Other conditions of extraction were kept constant (extracting temperature 70°C and extraction time 1 hour). Each extract solution was centrifuged at 2000 g, for 10 min, filtered through Whatman filter paper grade No. 1, and concentrated to 10 mL at 80°C in hot air oven. These aliquots of extracts were filtered through a 0.45 *μ*m PVDF syringe filter prior to analysis of genistein by UPLC-APCI-TOF-MS. From each parameter study, the highest yielding factor is considered for further parameter optimization studies.

#### 2.6.2. Effect of Temperature on the Extraction Yield of Genistein

The temperature of extraction influences the molecular movement, and also, it plays a major role in the conversion of glycosides to aglycones [31]. Since genistein is an aglycone, it is necessary to determine at what temperature maximum conversion of glycoside (genistin) to aglycone (genistein) occurs during extraction. Extraction temperature was varied between 70 and 100°C to determine the optimum extraction temperature for genistein from sample, while all other conditions of extractions were kept constant. Further procedure is carried out same as mentioned in the previous parameter study.

#### 2.6.3. Effect of Time on the Extraction Yield of Genistein

To achieve quantitative recoveries, extraction time is a factor that would significantly influence the extraction efficiency of genistein from medicinal plants and seeds. A range of extraction times 1, 2, 3, and 4 hours was selected for the experiments, while all other conditions of extraction were kept constant. Further procedure is carried out same as mentioned in the previous parameter study.

### 2.7. Validation Parameters

The method was validated according to the International conference on harmonization guidelines [32]. The parameters considered for the validation include linearity, precision, accuracy, limits of detection, and quantification.

#### 2.7.1. Linearity

To ascertain the linearity, a standard solution of known concentrations (5-125 ng/mL) was prepared and was fed individually in triplicate to the UPLC system. The calibration curve was established by plotting peak area versus concentration where the square of the correlation coefficient *R*^2^ >0.99 is indicative of the measure of linearity.

#### 2.7.2. Precision

The precision of an analytical method is defined as the multiple aliquots of analyte yielding a same/close reading which is when measured repeatedly from a single homogeneous system. Relative standard deviation (% R.S.D.) application was used to determine the precision of the method. Three independent determinations at three different concentration levels (15, 30, and 50 ng/mL) were measured on the same day at different interval of time that gives intraday precision and were measured on the consecutive days that give intermediate precision.

#### 2.7.3. Accuracy

The accuracy of the method was determined by the application of the standard addition method. Genistein at three different concentration levels (*n* = 9) (15, 30, and 50 ng/mL) was added to sample extracts, and the recovery percentage was calculated.

#### 2.7.4. The Limits of Detection (LOD) and Quantification (LOQ)

The limit of detection and quantification was determined from the calibration curve of standard. LOD and LOQ values were determined according to equations (1) and (2).

(1) $$\begin{array}{c} \text{LOD}=3.3\times \left(\frac{\sigma }{S}\right), \end{array}$$

(2) $$\begin{array}{c} \text{LOQ}=10\times \left(\frac{\sigma }{S}\right), \end{array}$$

*σ* is the standard deviation of the response or standard deviation of y-intercepts.

*s* is the slope of the calibration curve.

## 3. Results

### 3.1. Screening and Quantification of Genistein

**Figure 1:**
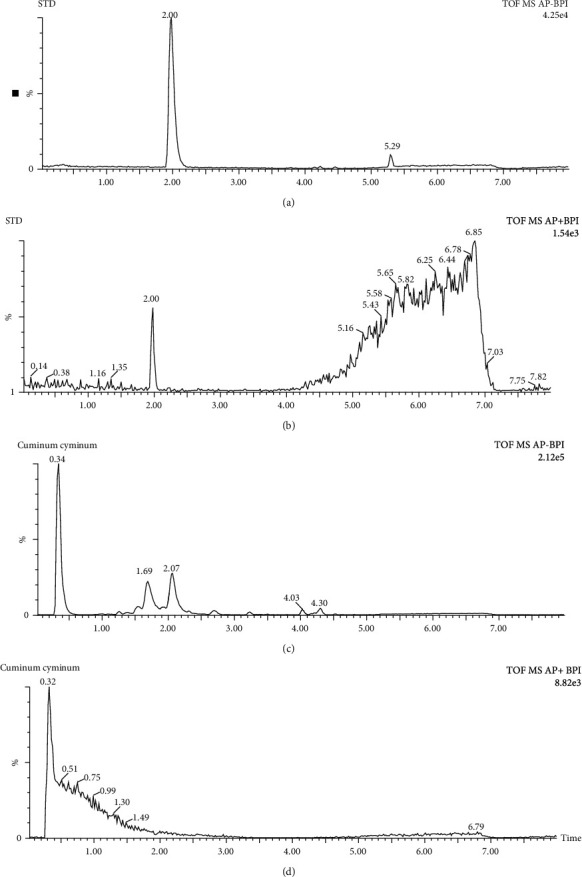
UPLC chromatogram of standard genistein and sample *Cuminum cyminum* ionized in negative mode (a, c) and in positive mode (b, d) by UPLC-APCI-TOF-MS, respectively.

**Figure 2:**
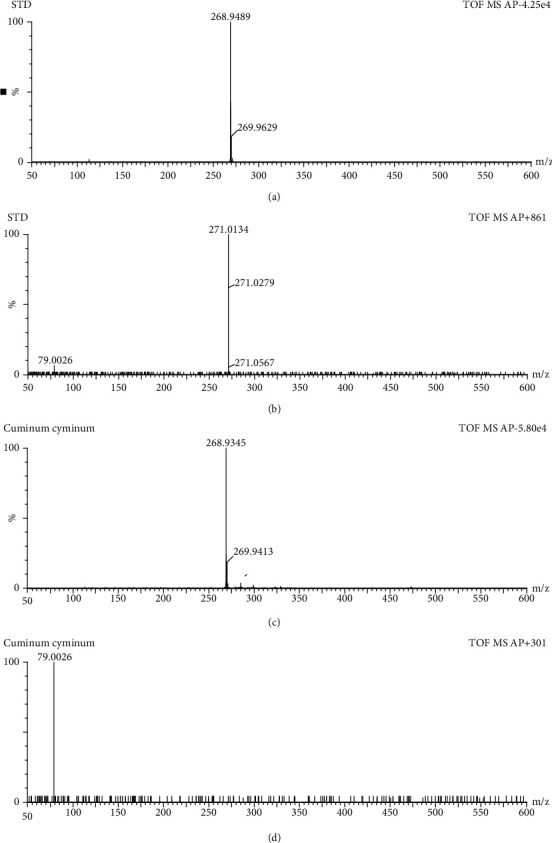
Mass spectrum of standard genistein and sample *Cuminum cyminum* ionized in negative mode (a, c) and in positive mode (b, d) by UPLC-APCI-TOF-MS, respectively.

**Figure 3:**
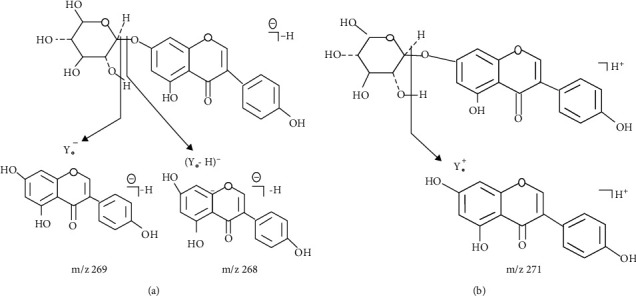
Fragmentation pattern of parent ion of genistein obtained by negative ion mode of UPLC-APCI-TOF-MS. (a) Fragmentation pattern of deprotonated glycoside to form free genistein. (b) Fragmentation pattern of protonated glycoside to form free genistein.

Seven different seed samples, prepared according to the extraction method explained above, were screened for genistein content using UPLC-APCI-TOF-MS coupled analytical system. Compounds separated by UPLC were ionized by APCI source, then are detected by TOF MS, and the results of which are represented as base peak intensity chromatogram (BPI). The presence of genistein in individual seed sample was confirmed by comparing retention time and mass spectra with that of the standard genistein. Among the seven varieties of seeds, the sample containing the highest concentration of genistein is considered for further studies. The quantification of genistein was done using the external calibration curve of the analytical standard. Only the *Cuminum cyminum* seed sample was found to contain genistein (226.67 *μ*g/g), and the remaining seed samples did not show any presence of genistein in the mass spectrum and chromatogram. Both negative and positive ion modes were tested for standard as well as for sample. Both standard and *Cuminum cyminum* samples were well ionized in the negative ion mode compared to positive ion mode (Figures 1 and 2). As shown in Figure 1, retention time (2.07 mins), obtained in UPLC chromatogram of sample *Cuminum cyminum*, was analysed by negative ion mode exactly matches with the retention time displayed by standard genistein under same method conditions. Furthermore, the presence of genistein in sample *Cuminum cyminum* is confirmed by mass spectrum obtained via mass spectrometer (Figure 2). The fragmentation pattern of the parent ion of genistein is shown in Figure 3. Sugar unit of deprotonated glycoside is cleaved to yield (Y_o_−H)^−^ ion of *m*/*z* 268 (Figure 3(a)) that forms the base peak in this product ion mass spectrum. At low collision energy, hydroxyl hydrogen atom undergoes rearrangement reaction and remains attached with the molecule to yield Y_o_^–^ ion of *m*/*z* 269 (Figure 3(a)). In general, the energy required for a rearrangement reaction is less compared to the scission process, and hence, the rearrangement reaction overtakes at low collision energies (∼15–30 eV). The loss of neutral unit from glucosyl group forms the Y_0_^+^ ion of *m*/*z* 271 (Figure 3(b)). Peak with retention time 2.07 minutes in sample chromatogram is identified as the analyte, genistein, and it was quantified as 220.82 *μ*g/g of *Cuminum cyminum*.

### 3.2. Optimization of Extraction Conditions

#### 3.2.1. Effect of Solvent Composition on the Extraction Yield of Genistein

**Figure 4:**
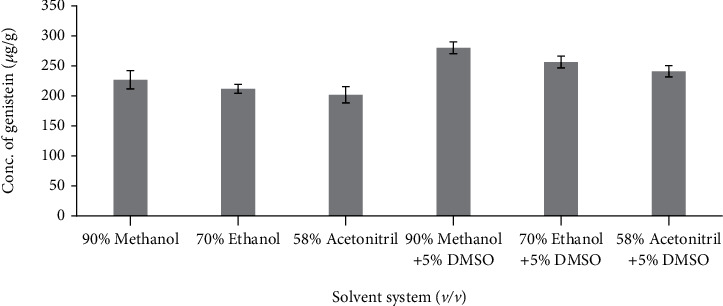
Influence of different solvent mixtures on *Cuminum cyminum* genistein content. (100 mL of solvent, temperature 80°C and time 1 h). (The bars show the mean values from three replicates (*n* = 3) with the respective standard deviations).

**Figure 5:**
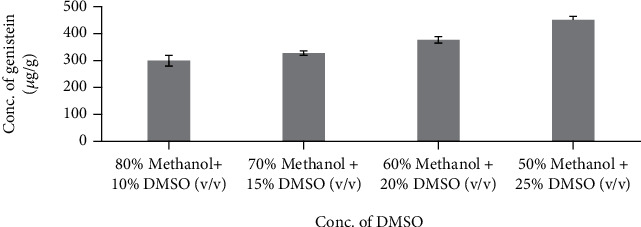
Influence of different concentration of DMSO on *Cuminum cyminum* genistein content. (100 mL of solvent (Methanol + DMSO), temperature 80°C and time 1 h). (The bars show the mean values from three replicates (*n* = 3) with the respective standard deviations).

The relative amount of genistein extracted from *Cuminum cyminum* seeds with different extraction solvent mixtures is shown in Figure 4. The extraction by the different solvent systems resulted in significant differences (*P* < 0.0001) among the concentrations of genistein and ranged from 201.67 *μ*g/g–280.03 *μ*g/g. The highest amount of genistein (280.03 *μ*g/g) was obtained with 90% Methanol and dimethyl sulphoxide solvent system. As shown in Figure 4, a solvent mixture consisting of dimethyl sulphoxide extracting highest amount of genistein compared to solvent mixture without dimethyl sulphoxide, and hence, study on concentration of dimethyl sulphoxide with the mixture of Methanol and water was conducted by varying the concentration of dimethyl sulphoxide from 5% to 25%.  Figure 5 shows that, with the increase in concentration of dimethyl sulphoxide, the concentration of genistein is also increasing correspondingly; this shows addition of dimethyl sulphoxide increases solubility of genistein present in *Cuminum cyminum* and extracts a remarkably high amount of genistein at a concentration of 25% from the sample compared to all other solvents alone. Concentration of dimethyl sulphoxide solvent beyond 25% was not considered in this study due to the high-temperature requirement for evaporation of dimethyl sulphoxide.

The maximum amount of genistein as measured by the UPLC peak areas was obtained when extraction was performed with Methanol : Water : Dimethyl sulphoxide (50 : 25 : 25, *v*/*v*/*v*), and hence, it was chosen as the best solvent for extracting genistein from *Cuminum cyminum* and used in the following experiments.

#### 3.2.2. Effect of Temperature on the Extraction Yield of Genistein

**Figure 6:**
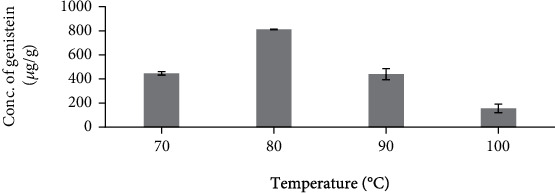
Influence of different temperature degree on *Cuminum cyminum* genistein content. (100 mL of solvent (50 mL Methanol+25 mL Dimethyl sulphoxide+25 mL Water (*v*/*v*/*v*)) and time, 1 h). (The bars show the mean values from three replicates (*n* = 3) with the respective standard deviations).

As shown in Figure 6, extraction at 70°C showed a relatively less amount of genistein compared to extraction at 80°C. Extraction at 90°C showed very less amount of genistein compared to extraction at 80°C and extraction at 100°C displayed the lowest amount of genistein compared to rest all extraction temperature. Extraction at a temperature less than 80°C falls insufficient to convert genistin to genistein present in sample and extraction at a temperature greater than 80°C causes degradation of genistein and hence extraction at 80°C proved to be the optimum extraction temperature for extracting genistein from the sample.

#### 3.2.3. Effect of Time on the Extraction Yield of Genistein

**Figure 7:**
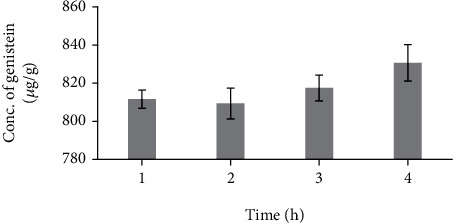
Influence of different time limits on *Cuminum cyminum* genistein content. (100 mL of solvent (50 mL Methanol+25 mL Dimethyl sulphoxide+25 mL Water (*v*/*v*/*v*) and temperature, 80°C). (The bars show the mean values from three replicates (*n* = 3) with the respective standard deviations).

To achieve quantitative recoveries, the time of extraction was increased from 1 to 2, 3, and 4 h using the optimized conditions (50 mL Methanol+25 mL DMSO+25 mL water at 80°C). As can be seen from Figure 7, all the genistein that could be extractable from the sample are extracted within 1 h and increasing extraction time beyond 1 h did not influence significant quantitative recovery, it has reached equilibrium. Therefore, extraction time of 1 hour was defined as the optimum time of extraction.

The efficiencies of the various solvents system, concentration of DMSO, temperature degrees, and time limits for the extraction of the genistein from *Cuminum cyminum* are summarized in Table 1. The extraction solvent, Methanol : Water : Dimethyl sulphoxide (50 : 25 : 25, *v*/*v*/*v*), temperature (80°C), and time (1 h) were found to be the ideal extraction conditions for extraction of genistein from *Cuminum cyminum.*

### 3.3. Method Validation

#### 3.3.1. Linearity

The linearity of the calibration curve of analytical standard genistein was constructed by plotting the area under the curve (AUC) of the main peak versus concentration. The calibration curve was found to be linear over the concentration range of 5–125 ng/mL with a correlation coefficient of 0.999.

#### 3.3.2. Precision

Both intraday and interday precisions of the analytical method were determined. In the intraday precision (*n* = 9) and interday precision analysis (*n* = 9), the values were found to be R.S.D. = 3.6% and R.S.D. = 4.8%, respectively. The obtained R.S.D. (%) values were lower than 5.0%, which attested the precision of the method.

#### 3.3.3. Accuracy

It was investigated by means of a standard addition experiment, at three concentration levels in triplicate (*n* = 9). The mean recovery of 107.23% (R.S.D. = 1.29%) assured the method accuracy.

#### 3.3.4. Limit of Detection and Quantification

The detection limit and quantification limit for genistein in UPLC MS/MS system were found to be 1.30 ng/mL and 4.45 ng/mL, respectively. The detection and quantification capacity of the analyte at a very low concentration level indicates high sensitivity of the method.

## 4. Discussion

Many plant products have been tested for genistein content from many years, including legumes, cereals, vegetables, fruits, and nuts. Legumes are known to be good sources for genistein as they contain a significant amount of genistein than other plant-based sources. Early study on phytoestrogen content of legumes reported a remarkably high amount of genistein in *Psoralea corylifolia*/Indian bread root (1528 *μ*g/g). Other legumes such as *Pueraria lobate/*kudzu, Lupines luteus/lupin, *Phaseolus vulgaris*/black turtle bean, *Phaseolus lunatus*/baby lima bean), *Phaseolus lunatus*/large lima bean, *Phaseolus vulgaris*/red kidney bean, and *Glycine max*/Soybean were reported to have 316 *μ*g/g, 86.7 *μ*g/g, 45.1 *μ*g/g, 40 *μ*g/g, 34.4 *μ*g/g, 29.3 *μ*g/g, and 24.1 *μ*g/g, respectively [33].

A variety of cereals have been screened for genistein content by Liggins et al., 2002. They found Wholegrain brown rice and shredded wheat to have 0.5 *μ*g/g of genistein but less than 1 *μ*g/g, whereas White rice, Corn flour, and Maize meal have 10 ng/g but less than 150 ng/g [34]. Later study on Quinoa grains found to contain a very minimal amount of genistein than other reported cereal grains, i.e., 2.5 pg/g only [35]. Genistein content in coffee and espresso coffee at different roast degrees has been estimated to vary between 0.2 *μ*g/g and 5.1 *μ*g/g [36].

Kuhnle et al., 2009, had conducted research to find out the concentration of genistein in commonly eaten vegetables and fruits in the U.K. They have found that fruits such as Apple, Banana, Apricot, Gooseberry, Lemon, Kiwi, Lychee, Mango, Watermelon, Papaya, Passion fruit, Peach, Pear, and Avocado have less than 1 *μ*g/100 g. Fig (dried), Grape (black, seed removed), and Grape (red, seedless) contained 12 *μ*g/100 g, 4 *μ*g/100 g, and 3 *μ*g/100 g, respectively. Berries and currants like Blackberries, Raspberries, Cranberries, and Red currant were shown to have less than 1 *μ*g/100 g. With respect to vegetables, a variety of vegetables in both raw and cooked form were tested for genistein content. Vegetables like Cabbage, Carrot, Radish, Cauliflower, Onion, Cucumber, Lettuce, Potato (boiled), Pumpkin, and Sweet corn (boiled) were reported to have less than 1 *μ*g/100 g. Beans and other vegetables have shown to have a considerable amount of genistein which includes Green bean (16 *μ*g/100 g), Red lentils (19 *μ*g/100 g), Parsley leaves (57 *μ*g/100 g), Butter beans (21 *μ*g/100 g), French beans (35 *μ*g/100 g), Haricot beans (14 *μ*g/100 g), Kidney beans (26 *μ*g/100 g), Runner beans (78 *μ*g/100 g), Bean sprouts (225 *μ*g/100 g), and Chickpeas (79 *μ*g/100 g). Among all the tested vegetables, Soybean and Soybean-derived foods have been found to contain the highest concentration of genistein and vary from 5540 to 13770 *μ*g/100 g [23]. The majority of the work carried out has been on Soybean and Soybean-based food products. The concentration of genistein in Soybean of different seed coat colour (yellow, green, and black) of six different genotypes have been found to vary from 117.7 to 942.2 mg/100 g dry weight [37]. Concentrations of genistein in species are influenced by genotypes, climate condition, agronomic practices, maturity at harvesting time, storage, and processing conditions [38, 39].

To date, only legumes have been the main source for genistein, of which *Genista tinctoria* [29], *Psoralea corylifolia* [33], and *Glycine max* [23] were found to contain the highest amount of genistein compared to all other variety of beans and other legumes. Only Soybean is found to be used regularly in the human diet, and other two species are found to be used as therapeutic molecule in traditional medicine rather than as a regular human food. This study explores a new nonleguminous source, *Cuminum cyminum*, a member of Apiaceae, containing 811.57 *μ*g/g of genistein as a novel food source of genistein. *Cuminum cyminum* has been identified as a therapeutic molecule from ancient time and reported to be beneficial against a variety of disorders including diabetes, cancer, and hypolipidemia. The *Cuminum cyminum* has been reported to have good inhibitory activity against *α*-glucosidase with IC_50_ value of 100 *μ*g/mL, whereas chemical drug, Acarbose, has an IC_50_ value of 25 *μ*g/mL suggesting the comparable efficiency of *Cuminum cyminum* against diabetes [40]. Hence, consumption of *Cuminum cyminum* in daily diet could serve beneficial against the development of various diseases.

Many chromatographic methods such as thin-layer chromatography (TLC)/high-performance thin-layer chromatography (HPTLC); HPLC with MS, UV, or PDA detector; and Gas chromatography-mass spectrometry (GC-MS) have been reported for quantification of genistein, but these methods were found to have poor resolution, lower sensitivity/selectivity, and longer run time, and due to this extended run time, methods demand more material resources. HPLC method employing ultraviolet (UV) detector led to improper separations of peaks and showed a poor range of linearity. LC combined with fluorescent detectors is limited as a number of isoflavones that are naturally fluorescent are rare and the acidification of the mobile phase has a quenching effect which in turn it reduces detector sensitivity [41]. GC-MS requires an extra derivatization step as genistein is not a volatile compound [28].

In order to facilitate high specificity and sensitivity, MS detection is a choice for the best detection system. Ultraperformance liquid chromatography (UPLC) coupled with MS system is one of the trending and preferred methods over other methods for detecting and quantifying analytes present as it is capable of providing the accurate retention time, high mass resolution, and UV-Vis absorption data for the identification. The elemental composition can be calculated with minimum (±5 ppm) mass error by the high-resolution mass. Electrospray ionization (ESI) and atmospheric pressure chemical ionization (APCI) are the two most commonly used ionization sources in mass spectrometry coupled liquid chromatography. Chen et al., 2002, analysed isoflavones content in soy nutrition supplement using HPLC-MS technique and compared ESI and APCI modes. A better separation and quantification with high sensitivity has been recorded with APCI mode for analysing isoflavones in soy nutrition supplement [42]. APCI mode was reported to be less susceptible to matrix effect than ESI. However, many quantification analyses have been carried out using ESI mode [43]. Sensitivity has been shown to increases up to 10-fold with UPLC and run time improvements have been found to be as large as 5-fold with good separation of peaks as compared to HPLC. Separation of diastereomers and peaks with narrower width possible with UPLC makes this technique superior with respect to resolution as compared to HPLC as HPCL technique fails to separate diastereomers [44]. Daems et al., 2016, reviewed 26 articles relevant to analytical methods used for quantification of isoflavones published between 2005 and 2015 and suggested that, for typical LC analysis, the approximate analysis time is between a minimum of 14 min and a maximum of 60 min [45]. In few studies using UPLC, analysis time has been found to be between 2.5 and 12 min [46–49]. These times could be further reduced by manipulating the column temperature, flow rate, mobile phase composition, and solvent gradients. For the first time, the UPLC-APCI-TOF-MS technique for quantification of genistein in the selected seeds of Apiaceae was successfully established with proper separation of peaks in only 8 mins of run time without any primary purification step. The developed method has the advantages of direct injection of sample for analysis without primary purification, increased sensitivity and specificity, and less time consumption compared to HPLC.

The information obtained above from the optimization study illustrate that solvent mixture (50 mL Methanol+25 mL Dimethyl sulphoxide+25 mL Water (*v*/*v*/*v*)), temperature (80°C), and time (1 h) are the best suitable condition for extracting genistein from *Cuminum cyminum*. The concentration of genistein before optimization was 226.67 *μ*g/g and after optimization is 811.57 *μ*g/g. This proves the efficiency of the method in the extraction of genistein without hydrolysis step, and the obtained optimized condition is in agreement with many other research works. Although the use of DMSO has been reported to have negative effects on chromatographic resolution [50], but in the current study, DMSO did not pose any such problems. In the case of other samples/instruments, the negative effects of DMSO can be resolved by precipitating genistein with the addition of water, and this precipitate can be used for analysis. In the case of large-scale productions, DMSO toxicity can be avoided by conducting extraction in closed reactors with automatic control and management system. As soon as the extraction is completed, genistein can be recovered through precipitation by the addition of water. Extraction of genistein by using DMSO increases the yield and subsequent precipitation by addition of water will be an efficient extraction technique for extraction of genistein from plant sources.

Study on comparison of extraction solvents used for the assay of isoflavones from Soybean shows correlating results by showing a high amount of genistein, when extraction is performed with the addition of Dimethyl sulphoxide [51]. Griffith and Collison, 2001, also documented the increase in extraction efficiency of soy isoflavones from 0.7 to 10.6% by the addition of Dimethyl sulphoxide [52]. Evaluation study conducted by Rostagno et al., 2007, found the optimum extraction conditions by Microwave-assisted extraction as 50°C, 20 min, and 50% ethanol for the extraction of isoflavones from Soybean. They found that, with the increase in temperature, malonyl and acetyl form of isoflavones convert into aglycone form [31]. The optimized condition for extraction of soy isoflavones by ultrasound-assisted extraction method were found to be 50% ethanol, 60°C reaction temperature, and 20 min of ultrasound-assisted extraction [53]. The Microwave and ultrasound-waves facilitate the rapid extraction of genistein compared to conventional extraction techniques. The optimum extraction conditions shown by Zhang et al., 2007 to extract soy isoflavones were found to be 80°C, 8 h with 96% ethanol [54]. Optimization study of genistein from Soybean conducted by Peñalvo et al., 2004, showed maximum recovery of genistein with 1 M HCl in 80% EtOH at 80°C within 0.5 h of the extraction time, and thereafter, no significant recovery occurred in the concentration of genistein with increase in extraction time. They found that extraction at 60°C to have no effect on conversion of glycoside to aglycone form, extraction at 70°C to be insufficient to convert all possible glycoside to aglycone, and extraction at 90°C and 100°C to produce less amount of genistein compared to extraction at 80°C due to degradation of genistein, which is again proved in this study that 80°C is the optimum extraction temperature for extraction of genistein from plant sources [55].

Based on these evidences and results obtained, it can be said that the composition of solvent and extraction temperature greatly influences the yield of genistein during extraction depending upon the type of plant material.

## 5. Conclusion

Diet is predicted to contribute to approximately 50% of cancers and other major chronic diseases in the advanced world. The incorporation of food rich in phytoestrogen in the human diet has proved to lower the risk of the development of several severe diseases including cancer, cardiovascular diseases, neurological disorders, and other degenerative diseases. In this study, *Cuminum cyminum* was discovered as a natural novel source for genistein, containing 811.57 *μ*g/g of genistein, and can be consumed in the regular human diet which increases wellness of human health along with enhancing the taste of the food.

Based on these studies, the concentration of DMSO and extraction temperature had the most profound effects on the extraction of genistein from *Cuminum cyminum*. Further research on isolation of genistein compound from the crude extract of *Cuminum cyminum* and *in vivo* and *in vitro* studies of genistein on various disease forms are essentials. A simple and easy method of extraction accompanied with rapid and highly sensitive UPLC-APCI-TOF-MS technique is successfully developed and validated. The system requires only 8 min of run time for complete separation of molecules and to display results. The high throughput, sensitive, and selective techniques facilitate the accurate quantification of many other potential isoflavones in complex matrices such as plant extracts without primary purification in a short duration.

## Figures and Tables

**Table 1a:** (a) Effect of solvent composition on the extraction yield of genistein

No.	Solvents	Concentration of genistein (*μ*g/g)
1	90% methanol	226.67 ± 1.68
2	70% ethanol	211.67 ± 0.64
3	58% acetonitrile	201.67 ± 1.62
4	90% methanol+5% DMSO	280.03 ± 0.76
5	70% ethanol+5% DMSO	256.25 ± 0.63
6	58% acetonitrile+5% DMSO	240.84 ± 0.73

**Table 1b:** (b) Effect of solvent composition on the extraction yield of genistein

No.	Concentration of DMSO	Concentration of genistein (*μ*g/g)
1	80% methanol+10% DMSO	299.40 ± 1.09
2	70% methanol+15% DMSO	327.67 ± 0.57
3	60% methanol+20% DMSO	376.70 ± 0.65
4	50% methanol+25% DMSO	451.16 ± 0.59

**Table 1c:** (c) Effect of temperature on the extraction yield of genistein

No.	Temperature (°C)	Concentration of genistein (*μ*g/g)
1	70	445.67 ± 0.53
2	80	811.23 ± 0.73
3	90	439.97 ± 1.54
4	100	155.6 ± 1.19

**Table 1d:** (d) Effect of time on the extraction yield of genistein

No.	Time (h)	Concentration of genistein (*μ*g/g)
1	1	811.57 ± 1.02
2	2	809.67 ± 1.43
3	3	817.50 ± 1.45
4	4	830.60 ± 2.39

∗All data are presented as mean ± SD (*n* = 3).

## Data Availability

All the data are included in the manuscript.
